# Acute Unilateral Hypopyon Uveitis and Secondary Glaucoma in an Adult With Relapsing Acute Lymphoblastic Leukemia

**DOI:** 10.7759/cureus.24968

**Published:** 2022-05-13

**Authors:** Mithun Thulasidas, Sagarika Patyal

**Affiliations:** 1 Glaucoma, Centre for Sight, New Delhi, IND; 2 Glaucoma, Centre For Sight, New Delhi, IND

**Keywords:** acute lymphoblastic leukemia (all), relapse, leukemia, secondary glaucoma, hypopyon uveitis

## Abstract

Anterior segment infiltration in acute lymphoblastic leukemia (ALL) presenting as hypopyon uveitis in an adult is rare. We report this case as an uncommon presentation in a patient in remission after chemotherapy for ALL. In addition to the hypopyon, the patient presented with congested eye caused by secondary raised intraocular pressure. There is a need to maintain a high index of clinical suspicion in uveitis cases, as early diagnosis of ocular malignancy can save vision. Atypical unilateral hypopyon, even in adults, can be an indication of relapsing ALL.

## Introduction

Ocular involvement is a well-documented complication of leukemia [1]. However, it is rare for ocular findings to be the initial manifestation of a new or relapsed disease [2]. Ocular involvement may occur by direct infiltration of neoplastic cells, hemorrhage, or ischemic changes [3]. It can manifest as intraocular inflammation not responding to conventional therapy, referred to as masquerade syndrome [4].

Primary retinal lymphoma presenting as uveitis in a quiet eye in adults is the most frequent cause of masquerade syndrome [5]. Hypopyon uveitis, although rare, has been reported in relapses of leukemia. Anterior segment involvement in acute lymphoblastic leukemia (ALL) is typically bilateral, occurs in 2.5% to 18% of cases, and almost all fall in the pediatric age group [6]. Here, we describe an adult patient presenting with unilateral leukemic hypopyon uveitis and secondary glaucoma as an initial sign of ALL relapse.

## Case presentation

A 24-year-old male patient presented with sudden onset of diminution of vision, pain, redness, and watering in the left eye (LE) for a week. He was treated elsewhere as a case of steroid-induced glaucoma and was on oral acetazolamide thrice a day and topical anti-glaucoma medications (timolol maleate 0.5%, dorzolamide hydrochloride 2%, brimonidine tartrate 0.2%) in LE for six days. Two years earlier, he was diagnosed with ALL, received chemotherapy, and the maintenance phase was completed three months ago, with successful remission.

**Figure 1:**
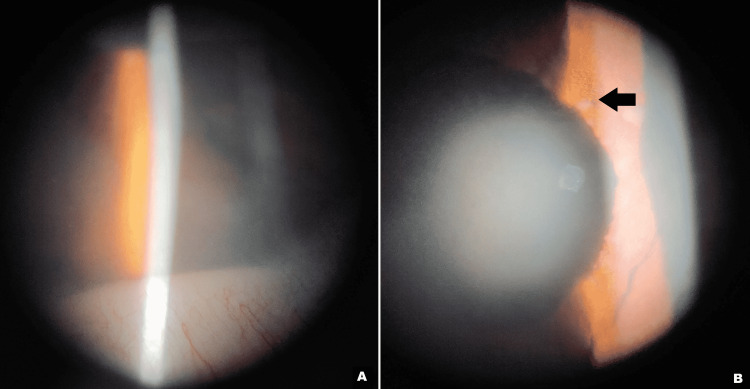
Anterior segment photograph showing (A) blood-streaked hypopyon and (B) nodular iris infiltrates (black arrow).

The corrected distance visual acuity (CDVA) in the right eye (RE) was 20/20 and in the LE was 20/80. The RE examination was unremarkable. The LE showed conjunctival and ciliary congestion, corneal epithelial edema with a few keratic precipitates on the endothelium, 2+ cells and moderate flare in the anterior chamber (AC) with irregular depth and blood-streaked hypopyon, thickening of iris stroma with multiple nodular infiltrates, sluggishly reacting pupil, and a clear lens (Figure 1).

Applanation intraocular pressure (IOP) was 14 mmHg in the RE and 34 mmHg in the LE. Fundus examination was within normal limits in both eyes.

Considering the clinical picture of non-resolving hypopyon and iris infiltrates, as well as the systemic history of the patient, masquerade syndrome was suspected. He was prescribed topical steroids (1% prednisolone acetate), cycloplegics, and anti-glaucoma medications were continued. A complete blood profile was done. However, the report was within the normal range. Moreover, the bone marrow aspiration done a few days ago showed no evidence of malignant cells. Nevertheless, the patient was advised to repeat the bone marrow aspiration and biopsy and was referred to the oncologist. Interestingly, the repeated bone marrow aspiration and biopsy showed 45% blasts indicating a relapse of ALL. Cerebrospinal fluid analysis for malignant cells was negative. He received hyper CVAD (cyclophosphamide, vincristine, adriamycin, and dexamethasone) chemotherapy as per protocol uneventfully.

**Figure 2:**
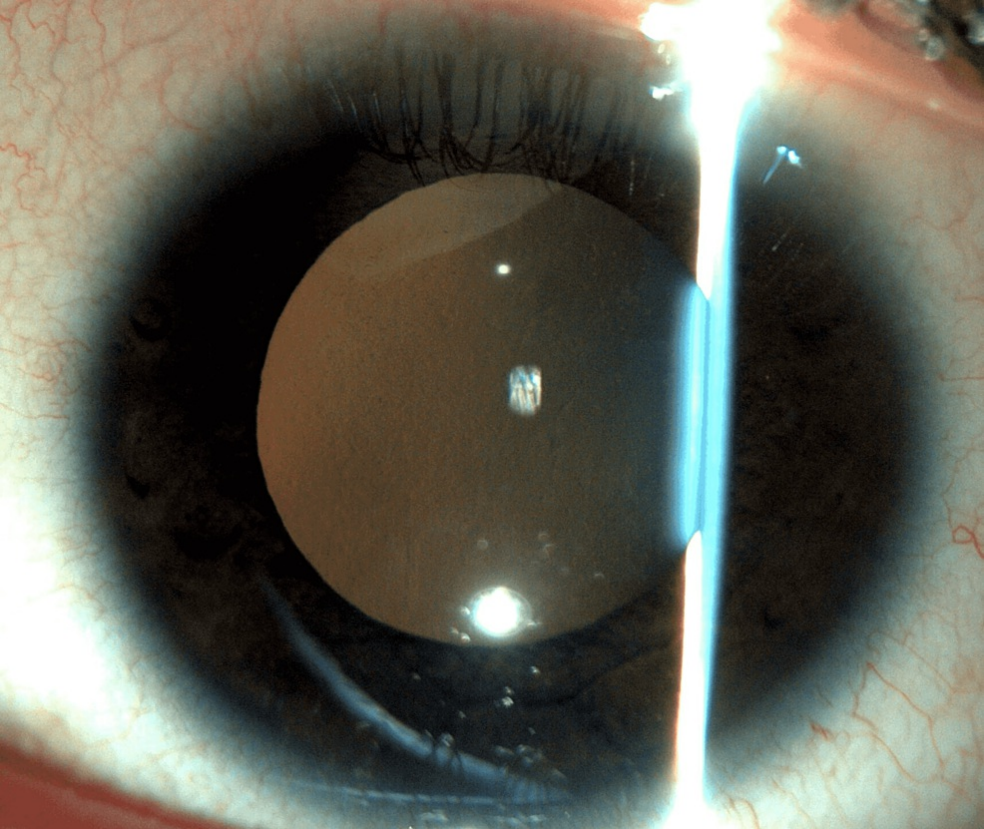
Anterior segment photograph showing a quiet eye with the resolution of hypopyon and iris infiltrates.

At the one-month follow-up, the LE CDVA was 20/20 with an IOP of 10 mmHg. The hypopyon and the iris infiltrates disappeared (Figure 2).

At the three-month follow-up, the bone marrow aspiration and biopsy showed hypocellular marrow with only 2% cells. The LE CDVA was 20/20 with an IOP of 11 mmHg. The ocular examination was normal, and all ocular medications were stopped.

## Discussion

Ocular symptoms in leukemia can present after the systemic diagnosis, be the presenting signs of the disease, or be the first manifestation of relapse after remission, like in our case [7]. Ophthalmic involvement is classified into two major categories, namely, primary/direct leukemic infiltration, mostly seen in relapse, or secondary/indirect involvement, commonly seen in the early phase of diagnosis. Ophthalmic screening of leukemia patients at the time of presentation and routine examination every six months (or earlier if there are ocular complaints) is warranted [8]. Hypopyon uveitis associated with ALL relapse in adults has been reported previously in three cases (Table 1) [9-11].

**Table 1:** Previously published reports of hypopyon uveitis associated with ALL relapse in adults. ALL: acute lymphoblastic leukemia

Author, year	Age of the patient	Presentation	Treatment
Gruenewald et al. (1979) [9]	37 years	Conjunctival congestion, iritis with diffuse ciliary flush, anisocoria, and presence of red blood cells with a fibrin-proteinaceous material and hypopyon in the anterior chamber	Topical and subconjunctival corticosteroids and local X-irradiation
Wetzler et al. (2000) [10]	64 years	Hyperemia of the sclera and hypopyon	Intrathecal chemotherapy with methotrexate, cytarabine, and hydrocortisone along with the use of topical glaucoma and corticosteroid drops
Yi et al. (2005) [11]	56 years	Corneal epitheliopathy with conjunctival injection and blood-streaked hypopyon	Topical corticosteroids and radiotherapy

In the present case, the patient had similar clinical signs (blood-streaked hypopyon with secondary glaucoma) as previously reported for hypopyon uveitis in leukemia relapse [9-11]. In addition, our patient had significant conjunctival and ciliary congestion attributable to raised IOP. An increase in IOP can occur due to the presence of tumor cells in the AC.

Molecular mechanisms by which leukemic cells give rise to extramedullary dissemination remain obscure. However, evidence suggests that chemokines may be involved in the organ-specific homing of neoplastic cells [12]. It is postulated that the blood-ocular barrier may be responsible for creating a “pharmacological sanctuary,” resulting in suppression of malignant cells, but not eradication by chemotherapeutic agents. These cells may be responsible for relapse [13].

Ocular involvement in patients with leukemia may be associated with a poorer prognosis. In a report by Matano et al., all patients died within one year of developing leukemic hypopyon [14]. Our patient also died within one year of this presentation.

## Conclusions

Clinicians should be aware that ocular involvement, even if unilateral, may be the sole early manifestation of leukemia relapse, even in adult ALL patients in the remission phase. Patients with hypopyon uveitis may present with or without the classical clinical signs of masquerade syndrome depending upon coexisting ophthalmic pathology. Unilateral hypopyon, especially if steroid-resistant, should prompt further action such as an AC tap for timely diagnosis. Early referral to a hemato-oncologist is crucial for evaluating multiorgan involvement and initiating proper treatment.
